# Fevers in Marquette County

**Published:** 1852-09

**Authors:** Ira Manley


ART. II. *Fevers in Marquette County, Wis.* By IRA MANLEY, Jr., M.D.

SIX years since, this county had scarcely been trodden except by the aborigines; and three years ago, when I came to the State, very little could be learned respecting the diseases of the county from practitioners who had been here previously. At this time there is no physician within ten miles who has been here as long as myself, therefore I trust due allowance will be made for a limited experience.

It may not be amiss to state that a residence of one year in the Holland Colony, Mich., had given me an opportunity to observe the peculiar characteristics of miasmatic fevers.

There a colony of five thousand Hollanders were being acclimated in a Michigan forest, with much marsh and swamp interspersed. Bilious disease in its most severe form, congestive and remittent fevers, dysentery, &c., prevailed to a most disheartening extent. I found that bleeding and cold effusion would relieve congestion, and large doses of quinine would prevent its return.

With such an initiation to the West I came to the rolling prairies and bracing climate of this county, in the spring of 1849, in search of a *healthier* location. So far from being a sickly season, there was an almost entire exemption from disease during the summer. The few cases which came under my notice inclined to a continued form. My remedies seemed to have but little effect; at least so far from proving abortive to the fever, I thought the patients would have been as well without the remedies. The cases were mild bilious remittent.

Early in the season of 1850 fevers began to appear of a very grave character. I will give the outlines of one of the first cases:

In conjunction with my friend, Dr. Walker, I attended M., æt. 28, who had just returned from Oshkosh, where he had what was termed typhus fever. After recovery he relapsed, in which condition he was sent home. I found him with dull expression of countenance, some cough, dullness over right lung, mucous rale, fever remittent. Treatment—alterative, expectorant; quinine in remission of fever; blisters externally. Patient died.

Autopsy—Both lungs extensively adherent; adhesions new at apex of right lung; right lung engorged. Spleen considerably increased in size, with an open abscess in one edge. Greater omentum occupying a very small compass above the transverse colon. No tenderness or tendency to tympanitis at this point during life.

About this time I heard of a number of fatal cases, in another part of the county, of what was called typhoid fever, in which I was sure quinine was largely used.

Case 2. M. J. G., about the age of first patient; habit spare. Complains of pain in the back and general uneasiness. Tongue lightly covered with white fur. Ordered a mild cathartic. After two days summoned again. Pain in the back has returned. J. says he cannot breathe. Pulse remarkably full and corded. Physical signs in lungs perfectly healthy. Tongue dry in centre, edges turned up. Bled patient; gave a large Dover's powder; bowels to be moved; bicarb. soda and nit. potas. combined in solution to be given every four hours. Left three powders of quinine grs. viij each, to be given when patient gets cool.

After taking two of the latter he sent for me. The conjestion had returned more violently than before. Pulse as before. Applied cloths wrung from hot water; gave a diaphoretic powder, and finally, the symptoms appeared so urgent, I bled again, which relieved him.

Left a powder of 10 grs. quinine, with directions as before. It was given in the night, soon after which I was hurried to his bedside again. I had already exceeded ordinary rules in venesection—my patient seemed in danger of immediate suffocation—I was almost at my wits' end. The pulse retained the same intensely corded feel. To be brief, I bled the third time! gave as many as 10 grs. of ipecacuan., applied the hot cloths vigorously. This treatment relieved the patient.

At each threatened return of the congestion, the large diaphoretic powder with the external applications and the continued use of the solution were the means which apparently saved the patient. The pulse remained full, and the patient recovered without any further exhibition of tonic or stimulant medicine. The fever continued about fifteen days before rapid recovery commenced.

I used but little quinine during the remainder of the season, except in two or three cases of persons who had contracted the disease in the timbered land towards Milwaukee.

I have never seen a case of ague here not imported from a distance.

You will imagine it was not without a severe mental struggle that I gave up my strong confidence in the abortive method of treating disease, having the prejudice in common with others in the country, that an eastern education does not fit a medical man to combat western diseases. The writers of articles in your journal, which at that time I had the privilege of reading, were residents of districts where miasma greatly influences the development of disease. If I called a counsel the first question would be, Have you prescribed quinine? Such being the case, I left the abortive treatment with trembling, and not till I was forced to do so by repeated experiment. I was so fortunate as to lose no case but the first one reported during the season.

There are but few cases where venesection may not be dispensed with, but I never have had occasion to regret the use of the lancet. I find the use of water, cold or hot, generally obviates its necessity.

In the part of my ride in the vicinity of Fox river there are extensive marshes, but I do not observe any difference in the amount or character of disease in this neighborhood. The river is ten miles north-west. Sixteen miles south-east on the head waters of Rock river, at Waupun, three very intelligent physicians reside. The country is level and much of it heavily timbered. They inform me that they use more than an ounce of quinine a week, yet fevers with them are assuming a decidedly continued form, very few cases being shortened by the abortive method. I need not particularize the details of my treatment. I have prescribed but little calomel, but have used alkalies freely,—commonly bicarb. soda and nit. potas., combined in solution,—a practice pointed out to me when a student by my preceptor, and found described in *Braithwaite's Retrospect.* I confess the rationale is made much more comprehensible to me by Prof. Herrick's explanation of the physiology of the liver, (New Series, Vol. 1, No. 1, p. 40, *N. W. Med. and Surg. Journal.*)

My treatment is what will be termed antiphlogistic. Some cases receive but a single prescription, but the most of them continue under treatment from six to fourteen days.

Condie's principles, in note to Watson's continued fever, have been my guide in the bilious remittent fever here. Very likely seasons will occur in which I shall have to use quinine.

I claim no originality, nor do I write with the view of making rules for others, but I would wish most earnestly to impress on young gentlemen the sentiment our teachers so much dwell upon, viz.: Let every man *think* and judge for himself, and whether a northern or southern man, remember that a difference in distance of but few miles may make a very material pathological difference.

There has been frequent reference in this part of the country to a typhoid fever, but I have never seen such a condition except in cases which were improperly managed. My observations have been confined to the southern part of Marquette county, a part of Fond du Lac, and the northern parts of Dodge and Columbia counties.

